# Risk stratification models for predicting preventable hospitalization in commercially insured late middle-aged adults with depression

**DOI:** 10.1186/s12913-023-09478-5

**Published:** 2023-06-13

**Authors:** Lauren Evans, Yiyuan Wu, Wenna Xi, Arnab K. Ghosh, Min-hyung Kim, George S. Alexopoulos, Jyotishman Pathak, Samprit Banerjee

**Affiliations:** 1 Division of Biostatistics, Department of Population Health Sciences, Weill Cornell Medicine, 402 East 67th Street, New York, NY 10065 USA; 2 Division of General Internal Medicine, Department of Medicine, Weill Cornell Medicine, 350 Ladson House 70th St, New York, NY 10065 USA; 3 Division of Health Informatics, Department of Population Health Sciences, Weill Cornell Medicine, 425 East 61st Street, New York, NY 10065 USA; 4 Weill Cornell Institute of Geriatric Psychiatry, Weill Cornell Medicine Psychiatry, 21 Bloomingdale Rd, White Plains, NY USA

**Keywords:** Emergency department, Preventable hospitalization, Service utilization, Risk adjustment, Late middle-aged, Depression

## Abstract

**Background:**

A significant number of late middle-aged adults with depression have a high illness burden resulting from chronic conditions which put them at high risk of hospitalization. Many late middle-aged adults are covered by commercial health insurance, but such insurance claims have not been used to identify the risk of hospitalization in individuals with depression. In the present study, we developed and validated a non-proprietary model to identify late middle-aged adults with depression at risk for hospitalization, using machine learning methods.

**Methods:**

This retrospective cohort study involved 71,682 commercially insured older adults aged 55–64 years diagnosed with depression. National health insurance claims were used to capture demographics, health care utilization, and health status during the base year. Health status was captured using 70 chronic health conditions, and 46 mental health conditions. The outcomes were 1- and 2-year preventable hospitalization. For each of our two outcomes, we evaluated seven modelling approaches: four prediction models utilized logistic regression with different combinations of predictors to evaluate the relative contribution of each group of variables, and three prediction models utilized machine learning approaches - logistic regression with LASSO penalty, random forests (RF), and gradient boosting machine (GBM).

**Results:**

Our predictive model for 1-year hospitalization achieved an AUC of 0.803, with a sensitivity of 72% and a specificity of 76% under the optimum threshold of 0.463, and our predictive model for 2-year hospitalization achieved an AUC of 0.793, with a sensitivity of 76% and a specificity of 71% under the optimum threshold of 0.452. For predicting both 1-year and 2-year risk of preventable hospitalization, our best performing models utilized the machine learning approach of logistic regression with LASSO penalty which outperformed more black-box machine learning models like RF and GBM.

**Conclusions:**

Our study demonstrates the feasibility of identifying depressed middle-aged adults at higher risk of future hospitalization due to burden of chronic illnesses using basic demographic information and diagnosis codes recorded in health insurance claims. Identifying this population may assist health care planners in developing effective screening strategies and management approaches and in efficient allocation of public healthcare resources as this population transitions to publicly funded healthcare programs, e.g., Medicare in the US.

**Supplementary Information:**

The online version contains supplementary material available at 10.1186/s12913-023-09478-5.

## Introduction

Patients with depression often develop chronic medical illnesses, including cardiovascular disease, diabetes, and chronic obstructive pulmonary disease at an earlier age than their non-depressed counterparts [1]. The reasons are complex, and include health factors such as poor diet, poorer adherence to self-management regimens, obesity, sedentary lifestyles and smoking, as well as physiologic abnormalities occurring during depression, including high corticosteroid levels, pro-inflammatory states, and other metabolic factors [1]. It is well-established that there is a bidirectional relationship between depression and chronic illness, where chronic medical illnesses increase the likelihood of developing depression, and factors such as health-related distress, functional impairments, and symptom burden associated with these conditions may worsen depression [1].

Improving healthcare outcomes in late middle-aged adults with depression (aged between 55 and 64 years) is a priority area because during this time chronic conditions such as cardiovascular disease, stroke, and cancer often become apparent [2–5]. Patients with chronic medical conditions and comorbid depression have high numbers of hospitalizations, frequent emergency hospital admissions, long hospital stays, high risk of readmission, utilization and cost of general medical services, poor adherence to self-care regimens, [1, 6–11] and a tendency to experience greater somatic symptom burden [1, 12]. Late middle-aged adults also face unique social and health-related factors. In addition to chronic medical illness, they or members of their household begin to plan for retirement, a stressful life transition that may affect mental well-being [13]. Perceived poor health status has been found to be a predictor of loneliness in late middle-aged adults [14]. In the U.S., late middle-aged patients are approaching the age of eligibility for the publicly funded Medicare insurance program. Identifying high-risk individuals in this age group with chronic diseases and co-morbid depression has the potential to improve their healthcare trajectories and reduce costs through efficient allocation of healthcare resources and targeted care management programs [15, 16].

In addition to reducing costs, preventing hospitalization earlier in the life course can improve patient experiences [17, 18]. Hospitalization is not without risks. Complications from diagnostic and therapeutic procedures, reactions to therapeutic drugs, hospital acquired infections, [19–21] and functional decline are all risks of hospitalization [22–25]. Efforts to identify patients at risk for hospitalization have largely focused on re-hospitalization within several weeks after admission, rather than a first or future hospitalization within the next year or longer [26–29]. Predictive modeling of first instances of preventable hospitalization among patients with depression represents a promising avenue for identifying patients who may be at high risk for adverse health outcomes.

This study sought to demonstrate the feasibility of identifying late middle-aged adults with depression who are at increased risk of hospitalization. Specifically, we developed and validated predictive models of risk of preventable hospitalization (1-year and 2-year risk) in late middle-aged adults with depression. We used commercial insurance claims that are national in scope from four of the largest insurers in the U.S. – Aetna, Humana, Kaiser Permanente, and UnitedHealthcare. We used the diagnostic categories used in the CMS HCC risk adjustment system [30] together with the Psychiatric Case-Mix System (PsyCMS) developed in the Veterans Affairs health system, [31] as well as demographic characteristics, and prior healthcare utilization measures to capture health status and prior healthcare utilization of the patient population. Given the lack of access to care in many rural areas and the complex ways that sex influences chronic and mental health conditions, [32, 33] we examined how the relationship between chronic and mental health conditions and preventable hospitalization varied by sex and rural/urban residence.

## Methods

### Data source

This retrospective cohort study used claims data from the Health Care Cost Institute (HCCI) [34]. The HCCI data include de-identified claims from four of the nation’s largest health insurers (Aetna, Humana, Kaiser Permanente and UnitedHealthcare) for U.S. residents of all 50 states. The Institutional Review Board of Weill Cornell Medicine approved this study. The informed consent was waived by Weill Cornell Medicine Institutional Review Board because it involved secondary data analysis using deidentified data. All methods were carried out in accordance with relevant guidelines and regulations.

#### Establishment of the study cohort

Enrollees in a commercial insurance plan who were aged 55–64 were considered for inclusion in our study sample. Additionally, enrollees were required to have continuous medical benefits coverage for at least 36-months from January 2011 through December 2013 to be included in the study sample to accurately capture their medical history and risk of hospitalization in the study period. The 36 months of the study period were split into (1) Year 1 (the “base year”); and (2) Year 2 (to capture the 1-year risk of preventable hospitalization); and (3) Years 2 and 3 combined (to capture the 2-year risk of preventable hospitalization). Enrollees were excluded if they had a hospice or nursing home claim in the base year or if they did not have at least one medical claim during the base year.

In order to identify enrollees with depression, we used a validated method for identifying depression using administrative data [35, 36]. We required at least one inpatient claim for depression, or two outpatient or two physician claims with a diagnosis of depression, or one outpatient or physician claim for depression plus at least one antidepressant medication fill during the base year. The following International Classification of Diseases, Ninth Revision, Clinical Modification (ICD-9-CM) codes were used to identify individuals with a depression diagnosis in the base year: 296.20, 296.21, 296.22, 296.23, 296.24, 296.25, 296.26, 296.30, 296.31, 296.32, 296.33, 296.34, 296.35, 296.36, 300.4, and 311. To identify whether enrollees had a prescription fill for an antidepressant medication during the base year, we searched available prescription drug claims for NDC codes associated with an antidepressant medication, using the HEDIS Antidepressant Medication Management list produced by the National Committee for Quality Assurance that was in effect during the enrollee’s base year.

The population of community-dwelling adults aged 55 to 64 with 36 months of continuous enrollment in a commercial insurance plan with diagnosed depression who met our inclusion criteria was 71,682.

#### Measures

All predictors of preventable hospitalization were measured in the base year only. Candidate predictors included demographic characteristics and prior healthcare utilization measures, as well as binary variables to indicate the presence of hierarchical condition category (HCC) and Psychiatric Case-Mix System (PsyCMS) conditions. Each of these measures is described in greater detail below.

#### Demographic characteristics

Due to the effect of sex in the course and development of medical and mental health conditions, we included a binary variable to capture sex in our models. A binary variable was used to indicate whether the individual is a resident of a metropolitan core-based statistical area, as defined by the U.S. White House Office of Management and Budget and using population counts collected in the decennial Census [37]. In this approach, Metropolitan Statistical Areas (MSAs) are defined as having a Core-Based Statistical Area (CBSA) with least one urbanized area with a population of at least 50,000. The MSA is comprised of the central county or counties containing the core plus adjacent outlying counties that have a high degree of economic integration with the core area, [37] where at least 25% of the population commute to or from the core urban area for work [38]. MSAs have also been used as a method for grouping hospitals, and they tend to be stable over time [38].

#### Prior healthcare utilization measures

Variables that capture prior healthcare utilization often improve model performance [29]. We included several dichotomous markers to capture prior healthcare utilization in the base year: any hospitalization, hospitalization for a psychiatric condition, any emergency department use, and emergency department use for a psychiatric condition.

#### Hierarchical condition categories (HCCs)

We used the condition categories defined in Version 12 of the CMS MA HCC model, as this version was in use for claims incurred during the study observation window. This version of the CMS HCC model contains 70 HCCs (A list of these HCCs can be found in Supplementary Table 1) [39]. Hierarchies are imposed among related condition categories so that a person is coded for only the most severe occurrence among related diseases. For unrelated categories, the HCCs are additive, where an individual may be coded for none, one, or multiple HCCs. Finally, the hierarchical versions of the conditions are used in predictions [40].

#### PsyCMS psychiatric condition categories

We used the 46 condition categories defined using the PsyCMS Case-Mix System (A list of these PsyCMS psychiatric condition categories can be found in Supplementary Table 2). The PsyCMS system uses hierarchies to reduce overlap among closely related diagnosis codes [31]. The hierarchies employed in PsyCMS reduce overlap among closely related diagnoses by assigning individuals to the single category most likely to drive mental health and substance use utilization [31]. These hierarchies were developed based on the clinical assessment of severity, medical diagnostic criteria, and greater specificity [31].

#### Study outcomes

The outcome variables of interest were the 1- and 2-year risk of having a preventable hospitalization. We defined preventable hospitalization using the ambulatory care sensitive conditions adapted from the Prevention Quality Indicators put forth by the Agency for Healthcare Research and Quality [17, 18]. Binary variables were created to capture whether enrollees experienced an inpatient hospitalization with a primary diagnosis for one of the following conditions: diabetes short-term complications, diabetes long-term complications, uncontrolled diabetes, lower-extremity amputation, chronic obstructive pulmonary disease or asthma, hypertension, heart failure, dehydration, bacterial pneumonia, urinary tract infection, congestive heart failure, and perforated appendix, with certain conditions (e.g., asthma in younger adults and low birth weight) not included in our measure as they do not apply to our adult population. A list of the ICD-9 diagnoses corresponding to these conditions is available through AHRQ) [41].

#### Data analysis

We developed four prediction models using logistic regression (Models 1 through 4), with different combinations of predictors to evaluate the relative contribution of each group of variables. Model 1 included demographics (sex and whether enrollee was a resident of a metropolitan CBSA) and variables to capture whether the enrollee experienced selected healthcare utilization events in the base year (any hospitalization, hospitalization for a psychiatric condition, any emergency department use, and emergency department use for a psychiatric condition); Model 2 included demographics and HCC conditions; Model 3 included demographics and PsyCMS conditions; and Model 4 included demographics in addition to HCC and PsyCMS conditions.

We then included all predictors and utilized various machine learning algorithms in our remaining three models (Models 5 through 7). Models 5 through 7 included all of the variables used in the previous four models (demographics, prior utilization, HCC conditions, and PsyCMS conditions), with the addition of two-way interactions sex with other all other predictors and metropolitan CBSA resident status with all other predictors. Model 5 utilized logistic regression with Least Absolute Shrinkage and Selection Operator (LASSO) penalty; [42] Model 6 utilized random forests (RF); [43] and Model 7 utilized gradient boosting machine (GBM) [44]. We chose two classes of ML algorithms: a regression-based method (LASSO) that models additive effects of predictors along with simple two-way interactions of predictors with sex and a marker for rural/urban residence; and methods based on decision trees (GBM and RF) which model complex higher order interactions involving predictors. The LASSO machine learning algorithm applies shrinkage to the coefficients in the model, with a penalty on the sum of the absolute magnitudes of the regression coefficients, giving better prediction accuracy due to reduced variance [45]. RF and GBM are similar in that both produce ensembles of tree learners, but with differences in how they arrive at the final aggregate tree learner. RF starts by generating multiple bootstrapped samples from the original data, then fits uncorrelated decision trees and combines them using a technique called bagging, whereas GBM combines simple prediction models to produce a complex aggregate model using boosting [46, 47].

The overall sample of 71,682 was split into two random groups, with 2/3 of the sample used as a training set for model development and the remaining as the test set to estimate prediction accuracy. Because of the low frequency of the observed outcomes, we randomly chose the nonevents to match the events 1:1 to create a balanced training set for each outcome. For the analysis involving the 1-year risk of preventable hospitalization, the training set included 1,462 beneficiaries and the test set included 23,893 beneficiaries; for analyses involving 2-year risk of preventable hospitalization, the training set included 2,362 beneficiaries and the test set included 23,894 beneficiaries. All predictors were binary and those that had a frequency ratio (i.e., the ratio of the frequency of the most common category and the other category) greater than 20 were removed from analysis because of the low information content in these predictors. All models were trained on the training set and the prediction accuracy (area under the receiver operating characteristics curve or AUC) was estimated on the test set. All logistic regression models were examined for calibration (agreement between predicted and observed frequencies) using the Brier score and visual inspection, then, if needed, re-calibrated coefficients were obtained in the training data. Among the machine learning algorithms, Model 5 (the LASSO model) had the highest AUC in the training set and a five-fold cross-validation in the training set was used to select the tuning parameter of the LASSO model that maximized the AUC.

## Results

In our sample, 1.5% experienced preventable hospitalization in the 1-year prediction window (n = 1,096), and 2.8% experienced preventable hospitalization within the 2-year prediction window (n = 1,974). Approximately 70% of the sample was female, and all were aged 55–64 during the observation period. Nearly 90% resided in a metropolitan core-based statistical area.

**Fig. 1:**
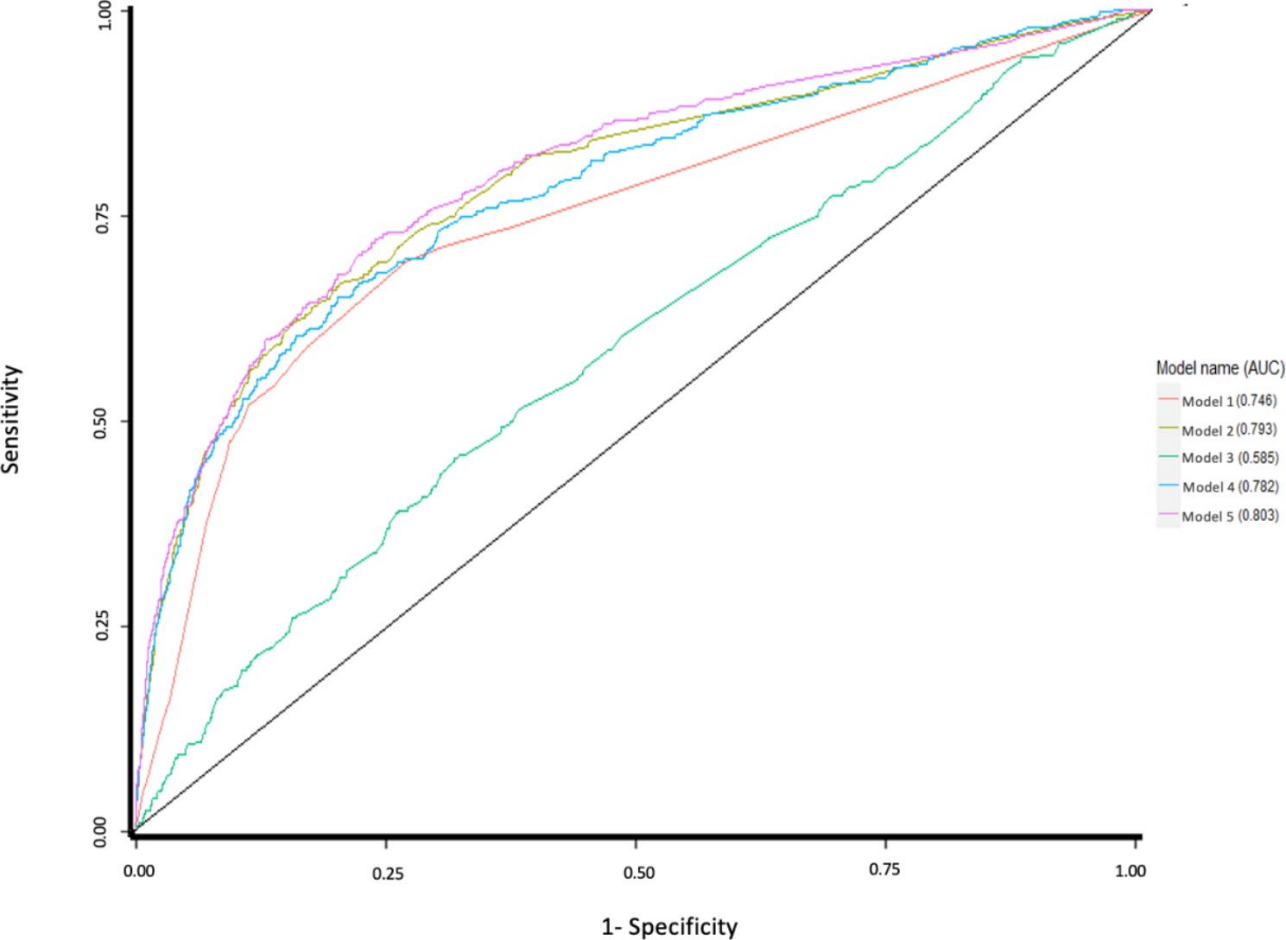
Receiver operating characteristic curves comparing accuracy of five models in predicting 1-year risk of preventable inpatient hospitalization Model Legend: Model 1 = Dem. + prior utilization Model 2 = Dem. + HCC Model 3 = Dem. + PsyCMS Model 4 = Dem., HCC + PsyCMS Model 5 = Dem., prior utilization, HCC, PsyCMS, interaction effects, with LASSO

**Fig. 2:**
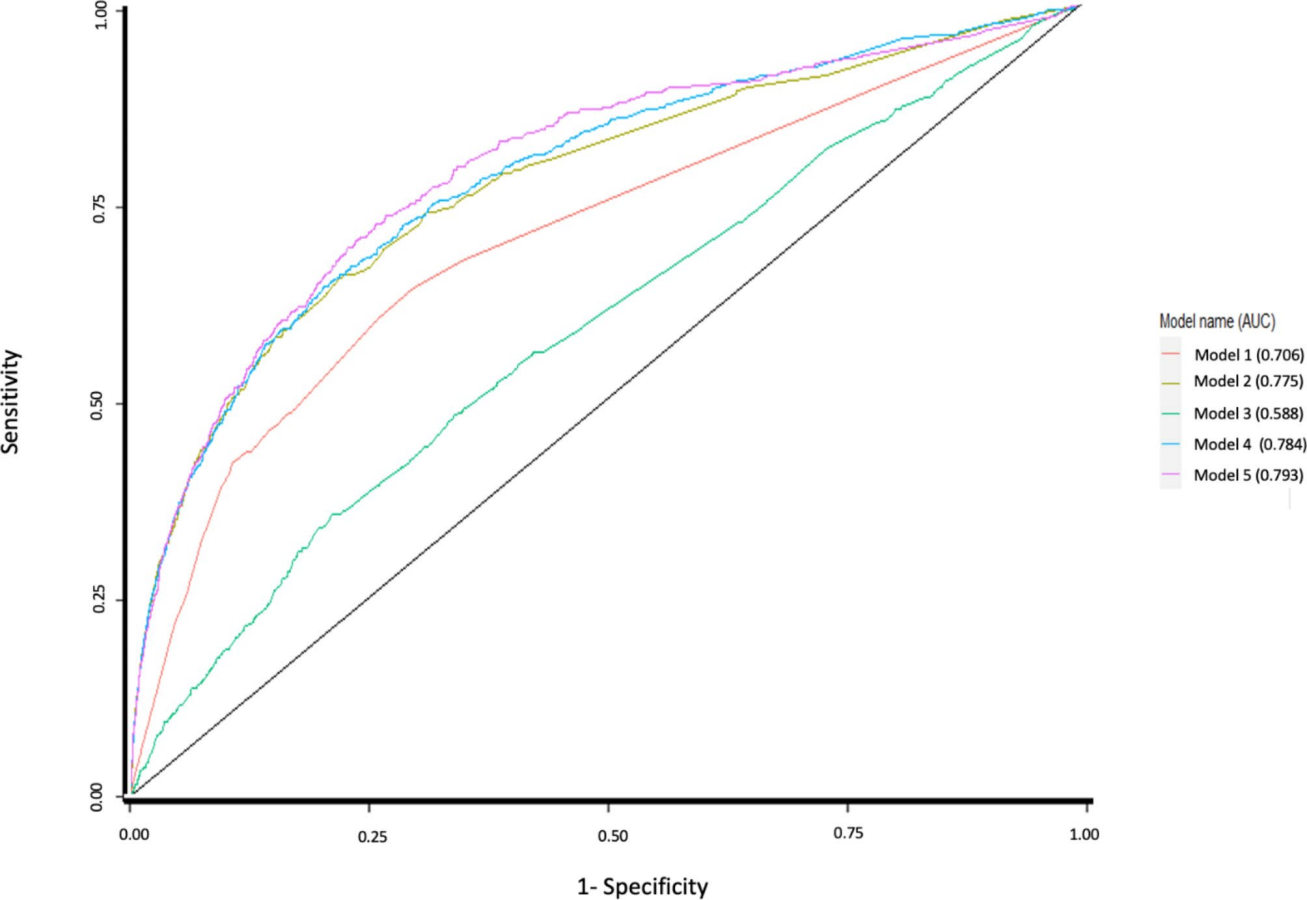
Receiver operating characteristic curves comparing accuracy of five models in predicting 2-year risk of preventable inpatient hospitalization Model Legend: Model 1 = Dem. + prior utilization Model 2 = Dem. + HCC Model 3 = Dem. + PsyCMS Model 4 = Dem., HCC + PsyCMS Model 5 = Dem., prior utilization, HCC, PsyCMS, interaction effects, with LASSO

In Table 1 we present a comparison of the model performance for the seven alternative modeling approaches for our two outcome measures, with corresponding Figs. 1 and 2 which show a graphical representation of the ROC curves. The sensitivity and specificity of our predictive models for 1- and 2-year risk of preventable hospitalization were obtained by choosing a threshold for the risk prediction functions that maximized the Youden index [48].

**Table 1:** Comparison of Prospective Model Performance Using Area under ROC Curve (AUC) for 1- and 2-year risk of Preventable Inpatient Hospitalization

	Model Variables used in Alternative Hospitalization Prediction Models*						
**Outcome†**	**Model 1 = Dem. and prior utilization**	**Model 2 = Dem. and HCC**	**Model 3 = Dem. and PsyCMS**	**Model 4 = Dem., HCC and PsyCMS**	**Model 5 = Final model‡ utilizing machine learning -** logistic regression with Least Absolute Shrinkage and Selection Operator (LASSO) penalty	**Model 6 utilizing machine learning –random forests (not shown in** Figs. 1 and 2**)**	**Model 7 utilizing machine learning –gradient boosting machine (not shown in** Figs. 1 and 2**)**
1-year risk of preventable hospitalization	0.746	0.793	0.585	0.782	0.803	0.718	0.765
2-year risk of preventable hospitalization	0.706	0.775	0.588	0.784	0.793	0.729	0.770

*All predictors and were derived from claims incurred during the base year

† 1-year prospective outcome data was measured using claims data from year 2, and 2-year prospective outcome data was measured using claims data from years 2 and 3 combined

‡ The Final model included all variables specified in other models plus interaction terms, after applying LASSO and filtering

Dem. indicates demographics; HCC indicates Hierarchical Condition Categories; PsyCMS indicates Psychiatric Case-Mix System conditions

For the one-year risk of preventable hospitalization, our best performing model was a machine learning model (Model 5, referred to as the Final Model in Table 1) with an AUC of 0.803. This model utilized the machine learning approach of logistic regression with LASSO penalty, and included demographic characteristics, prior healthcare utilization variables, HCC conditions, PsyCMS conditions, and interaction effects between sex and all other variables in the model and between the rural/urban indicator and all other variables in the model. For the 1-year risk of preventable hospitalization, using Model 5, a sensitivity of 72% and a specificity of 76% were obtained under the optimum threshold 0.453. Model 1 included only demographic variables and prior healthcare utilization variables and achieved an AUC of 0.746; Model 2 included demographic variables and HCC conditions (AUC = 0.793); Model 3 included demographic variables and PsyCMS conditions (AUC = 0.585); Model 4 included demographic variables, HCC conditions and PsyCMS conditions (AUC = 0.782); Model 6 utilized the machine learning approach of random forests and included demographic characteristics, prior healthcare utilization variables, HCC conditions, PsyCMS conditions, and all higher-order interactions (AUC = 0.718); and Model 7 utilized the machine learning approach of GBM and included demographic characteristics, prior healthcare utilization variables, HCC conditions, PsyCMS conditions, and all higher-order interactions (AUC = 0.765).

As for the 2-year risk of preventable hospitalization, we similarly found the best performing model was the one that utilized the machine learning approach of logistic regression with Least Absolute Shrinkage and Selection Operator (LASSO) penalty (AUC = 0.793) (Model 5, referred to as the Final Model in Table 1), which had a sensitivity of 76% and a specificity of 71% under the optimum threshold 0.452. It performed better than Model 1 (AUC = 0.706); Model 2 (AUC = 0.775); Model 3 (AUC = 0.588); Model 4 (AUC = 0.784); Model 6 (AUC = 0.729); and Model 7 (AUC = 0.770).

**Fig. 3:**
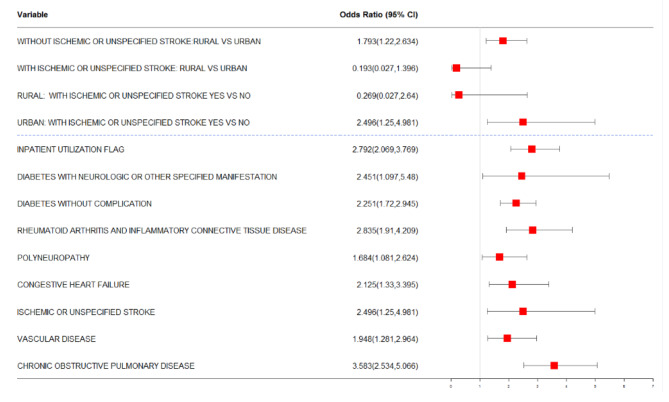
Risk factors identified by LASSO for 1-year risk of preventable hospitalization: The risk factors selected by the LASSO model (Model 5) are presented along with their Odds Ratio and 95% CI. Risk factors above the two dashed line correspond to the interaction between Ischemic or unspecified stroke and Rural residence. Marginal estimates of risk are presented for the four categories of the interaction effect adjusting for other risk factors in the model

**Fig. 4:**
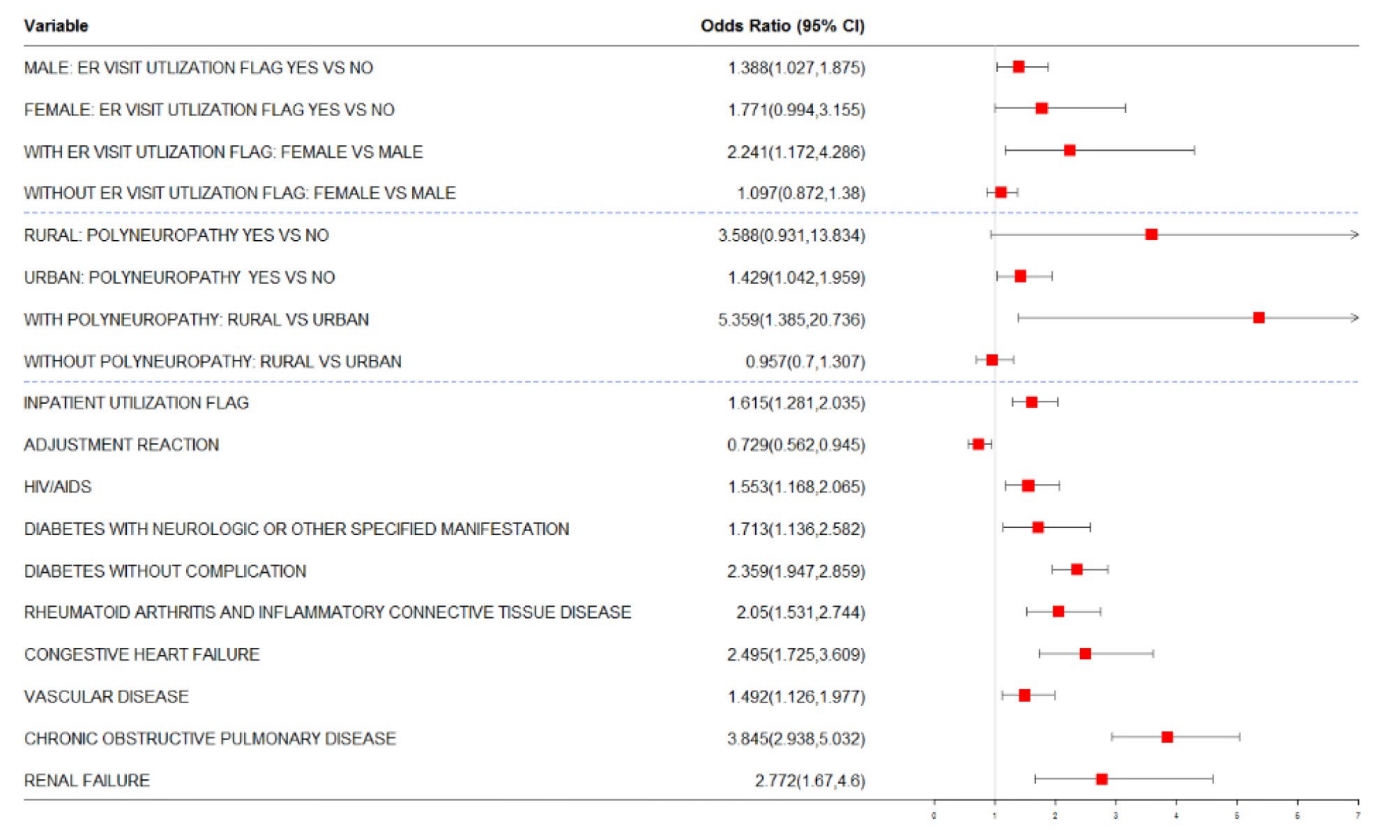
Risk factors identified by LASSO for 2-year risk of preventable hospitalization: The risk factors selected by the LASSO model (Model 5) are presented along with their Odds Ratio and 95% CI. Risk factors above the two dashed lines correspond to the interaction between Polyneuropathy and Rural residence and between Gender and ER utilization. Marginal estimates of risk are presented for the four categories of each interaction effect adjusting for other risk factors in the model

Our modeling approach allowed for the identification of main effects and interaction effects that influence the odds of preventable hospitalization. Figures 3 and 4 present risk factors identified for preventable hospitalization together with the odds of preventable hospitalization for 1- and 2-year risk of preventable hospitalization in our final models.

In our analysis involving the 1-year risk of preventable hospitalization, the following factors significantly increased the odds of preventable hospitalization: having an inpatient hospitalization in the base year, the presence of diabetes without complications, the presence of diabetes with neurologic or other specified manifestation, rheumatoid arthritis and inflammatory connective tissue disease, polyneuropathy, congestive heart failure, vascular disease, and chronic obstructive pulmonary disease (see Fig. 3). For the 1-year risk of preventable hospitalization, there was an interaction effect between ischemic or unspecified stroke and rural residence, with the marginal estimates of risk for the four categories of this interaction effect depicted in the top portion of Fig. 3. Among urban enrollees, ischemic or unspecified stroke increased the odds of preventable hospitalization, adjusting for other risk factors in the model.

In our analysis for the 2-year risk of preventable hospitalization, the following factors significantly increased the odds of experiencing a preventable hospitalization: having an inpatient hospitalization in the base year, having a diagnosis of HIV/AIDS, diabetes with neurologic or other unspecified manifestation, diabetes without complications, rheumatoid arthritis and inflammatory connective tissue disease, congestive heart failure, vascular disease, chronic obstructive pulmonary disease, and renal failure, and having a mental health diagnosis of adjustment reaction decreased the odds of preventable hospitalization (see Fig. 4). For the 2-year risk of preventable hospitalization outcome, significant interactions were observed between the rural/urban indicator and polyneuropathy and between sex and prior emergency department utilization, as depicted in the top portions of Fig. 4. Among individuals with prior emergency department use, females had an even higher odds of preventable hospitalization compared to males, and among individuals with polyneuropathy, rural residence increased the odds of preventable hospitalization.

## Discussion

We developed custom calibrated models to assess the feasibility of predicting preventable hospitalization in late middle-aged adults with depression. Our model development process relied on large set of clinical, demographic, and prior utilization variables that provided a rich description of the enrollees. The risk factors identified by our modeling approach are consistent with earlier literature [49–51]. This study demonstrates the feasibility of identifying late middle-aged adults with depression who are at high risk of hospitalization using data from health insurance claims.

We considered several machine learning algorithms for predicting both 1-year and 2-year risk of preventable hospitalization and our best performing was a logistic regression with LASSO penalty. The LASSO method performed better than the other candidate machine learning approaches of RF and GBM. This observation indicates that a regression-based model with additive effects of predictors along with hypothesized simple interaction effects of all predictors with sex and rural/urban residence sufficiently captures the variability in the two outcomes, without the need of including complex higher-order interaction effects by using the RF or GBM approaches.

Unsurprisingly, several chronic, prevalent comorbidities were strongly associated with high risk for preventable hospitalization, including diabetes, congestive heart failure, vascular disease and chronic obstructive pulmonary disease. In models for both 1- and 2-year risk of preventable hospitalization, diabetes (either without complications or with neurologic or other specified manifestations), rheumatoid arthritis and inflammatory tissue disease, congestive heart failure, vascular disease, chronic obstructive pulmonary disease, and a prior hospitalization in the base year, were risk factors for preventable hospitalization in late middle-aged adults with depression. These observations are consistent with previous literature [49–52]. Certain risk factors were associated with only 1-year risk of hospitalization (i.e., ischemic or unspecified stroke) and other factors were associated with only 2-year risk of hospitalization (i.e., adjustment reaction, prior emergency department use, HIV/AIDS, and renal failure). Our models revealed distinct differences in risk factors by sex and urban/rural residence. In particular, ischemic or unspecified stroke was associated with a high risk of 1-year preventable hospitalization in patients residing in urban settings. Polyneuropathy was associated with a high risk of 2-year hospitalization in rural patients. Having a history of prior emergency department visit in the base year was a risk factor for 2-year hospitalization for males.

This study demonstrates the feasibility of identifying groups of individuals among late middle-aged adults with depression who are at high risk of hospitalization. Algorithms, similar to ours, may serve to identify groups of individuals at high risk who may benefit from screening and services that can be proactively provided in outpatient settings [17, 18]. Such interventions include referrals to care management services, [53, 54] including care coordination, disease management programs, complex care management, disease-specific self-management education, health maintenance reminders, provider decision support tools, telephone support, and 24-hour consultation telephone lines [55]. A collaborative care model [56] may be another option for adults with chronic medical illness and depression at high risk of future hospitalizations. In this model, primary care and behavioral health services are integrated to address mental health and medical conditions concurrently [56]. Risk stratification algorithms have been used in other healthcare and led to successful interventions. Algorithms for identifying patients at risk for frailty have been used to target interventions that prevent, delay or treat frailty [57]. Similarly, algorithms have been used to identify individuals in need of assistance for emergency disaster preparedness [58].

A strength of our study is the use of insurance claims from several large U.S. insurers rather than insurance claims from a single insurer or geographic region. Our risk prediction models were based on diagnoses, prior utilization and demographic characteristics routinely captured in health records and do not rely on survey-based tools, screenings, or clinical assessments. Although information derived from clinical examination, patient interview, or medical record reviews serve important purposes for research investigations, data derived from claims databases are attractive because they are readily available and are less costly than other approaches [59]. Unlike other studies which assign diagnosis codes to diagnosis clusters known as Aggregated Diagnosis Groups (ADGs), [29, 60] our approach does not use proprietary algorithms that would make it difficult for researchers to fully explore data, and does not require a user license and a fee [29, 60].

Our study has several limitations. Our selection criterion of 36 months of continuous enrollment allowed us to take a long-term view of enrollees’ health outcomes. However, this selection criterion may limit the generalizability of our findings to other patient populations. Patients under the age of 65 years with continuous enrollment in commercial insurance are likely different than other patient populations, such as older patients enrolled in Medicare or patients younger than 65 years of age with interrupted employment that may be non-eligible for commercial health insurance. Another limitation is that diagnostic codes may change over time. For this reason, our findings require replication based on newer health insurance claims data. Future work may incorporate more detailed information involving sociodemographic characteristics, when available. Another limitation of our approach is that we relied on the claims’ coding process during the base year to identify the enrollees’ illnesses; in some cases enrollees may have a mental health or medical illness but not receive a diagnosis, and in other cases enrollees may be incorrectly diagnosed as having a condition.

In conclusion, this study demonstrates that our predictive modeling approach, using diagnoses, prior utilization and other demographic characteristics readily available in claims data, can be used to identify older adults with depression at high risk for preventable hospitalization. As the U.S. population ages, there is an increasing medical burden of individuals with depression. Our approach may assist health care planners in identifying various populations at risk for hospitalization that would be suitable for screening strategies and targeted referral processes and interventions to improve depression and chronic disease management.

## Electronic supplementary material

Below is the link to the electronic supplementary material.

Supplementary Material 1

## Data Availability

The datasets generated and/or analyzed during the current study are available from a third party (Health Care Cost Institute, HCCI) but restrictions apply to the availably of these data, which were used under license for the current study, and so are not publicly available. Data are however available from Samprit Banerjee upon reasonable request and with permission of Health Care Cost Institute (HCCI).
