# Supplementary material for: Risk stratification models for predicting preventable hospitalization in commercially insured late middle-aged adults with depression
Source: BMC Health Serv Res. 2023 Jun 13;23:621. doi: 10.1186/s12913-023-09478-5 (PMC10262395; doi:10.1186/s12913-023-09478-5)
Supplement: Supplementary file 1 — Supplementary Material 1 [file 12913_2023_9478_MOESM1_ESM.pdf]

**Supplementary Table 1: List of HCC Clusters**

HIV/AIDS
Septicemia/Shock
Opportunistic Infections
Metastatic Cancer and Acute Leukemia
Lung, Upper Digestive Tract, and Other Severe Cancers
Lymphatic, Head and Neck, Brain, and Other Major Cancers
Breast, Prostate, Colorectal and Other Cancers and Tumors
Diabetes with Renal or Peripheral Circulatory Manifestation
Diabetes with Neurologic or Other Specified Manifestation
Diabetes with Acute Complications
Diabetes with Ophthalmologic or Unspecified Manifestation
Diabetes without Complication
Protein-Calorie Malnutrition
End-Stage Liver Disease
Cirrhosis of Liver
Chronic Hepatitis
Intestinal Obstruction/Perforation
Pancreatic Disease
Inflammatory Bowel Disease
Bone/Joint/Muscle Infections/Necrosis
Rheumatoid Arthritis and Inflammatory Connective Tissue Disease
Severe Hematological Disorders
Disorders of Immunity
Drug/Alcohol Psychosis
Drug/Alcohol Dependence
Schizophrenia
Major Depressive, Bipolar, and Paranoid Disorders
Quadriplegia, Other Extensive Paralysis
Paraplegia
Spinal Cord Disorders/Injuries
Muscular Dystrophy
Polyneuropathy
Multiple Sclerosis
Parkinsons and Huntington’s Diseases
Seizure Disorders and Convulsions
Coma, Brain Compression/Anoxic Damage
Respirator Dependence/Tracheostomy Status
Respiratory Arrest
Cardio-Respiratory Failure and Shock
Congestive Heart Failure
Acute Myocardial Infarction
Unstable Angina and Other Acute Ischemic Heart Disease
Angina Pectoris/Old Myocardial Infarction
Specified Heart Arrhythmias
Cerebral Hemorrhage
Ischemic or Unspecified Stroke
Hemiplegia/Hemiparesis
Cerebral Palsy and Other Paralytic Syndromes
Vascular Disease with Complications

Vascular Disease
Cystic Fibrosis
Chronic Obstructive Pulmonary Disease
Aspiration and Specified Bacterial Pneumonias
Pneumococcal Pneumonia, Empyema, Lung Abscess
Proliferative Diabetic Retinopathy and Vitreous Hemorrhage
Dialysis Status
Renal Failure
Nephritis
Decubitus Ulcer of Skin
Chronic Ulcer of Skin, Except Decubitus
Extensive Third-Degree Burns
Severe Head Injury
Major Head Injury
Vertebral Fractures without Spinal Cord Injury
Hip Fracture/Dislocation
Traumatic Amputation
Major Complications of Medical Care and Trauma
Major Organ Transplant Status
Artificial Openings for Feeding or Elimination
Amputation Status, Lower Limb/Amputation Complications

**Supplementary Table 2: List of PsyCMS Clusters**

Dementia
Organic acute
Organic other
Alcohol dependence
Alcohol abuse
Alcohol use remission
Alcohol intoxication
Opioid dependence/abuse
Stimulant dependence
Stimulant abuse
Other specified drug dependence
Other specified drug abuse
Unspecified drug dependence
Unspecified drug abuse
Drug/alcohol withdrawal
Drug use remission
Nicotine dependence
Schizoaffective
Schizophrenia other
Paranoid schizophrenia
Psychoses not otherwise specified
Other psychotic disorders
Bipolar disorders
MDD severe
MDD recurrent
MDD single episode
Depression not otherwise specified/not elsewhere classified
Neurotic depression/dysthymia
Mood disorder not otherwise specified
Posttraumatic stress disorder
Panic/agoraphobia
Generalized anxiety
Anxiety not otherwise specified/not elsewhere classified
Personality disorders
Adjustment reaction
Conduct disorders
Major psychiatric disorder in remission
Other nonpsychotic
Pain
Sexual dysfunction
Sleep
Somatic other
Homeless MH problems
Jobless MH problems
Other MH V code
Pervasive developmental disorders
